# The Opinions of Poles about the Need to Provide Humanitarian Aid to Refugees from the Area Covered by the Russian–Ukrainian War

**DOI:** 10.3390/ijerph192013369

**Published:** 2022-10-16

**Authors:** Katarzyna Karakiewicz-Krawczyk, Krzysztof Zdziarski, Marek Landowski, Agnieszka Nieradko-Heluszko, Artur Kotwas, Paweł Szumilas, Anna Knyszyńska, Beata Karakiewicz

**Affiliations:** 1 Department of Specialist Nursing, Pomeranian Medical University in Szczecin, 71-210 Szczecin, Poland; 2 Subdepartment of Social Medicine and Public Health, Department of Social Medicine, Pomeranian Medical University, 71-210 Szczecin, Poland; 3 Department of Computer Science, Faculty of Computer Science and Telecommunications, Maritime University of Szczecin, 70-500 Szczecin, Poland; 4 Independent Research and Biostatistics Laboratory, Department of Social Medicine, Pomeranian Medical University, 71-210 Szczecin, Poland; 5 Department of Humanities and Occupational Therapy, Pomeranian Medical University, 70-103 Szczecin, Poland

**Keywords:** subjective feelings, help, refugees, war, Poles, Ukrainians

## Abstract

The armed conflict in Ukraine has caused a lot of emotions around the world. Many countries have been involved in helping brutally attacked people, especially mothers and children. The versatile involvement of large powers is monitored and noticeable by the media. Active help from Poland is a very visible sign of human solidarity with the suffering Ukrainian nation. Open hearts, houses and institutions and humanitarian and medical aid are elements of Polish kindness and empathy. The aim of the research was to collect Poles’ opinions about the need to provide humanitarian aid to Ukrainians from the territories of the Russian–Ukrainian war. Capturing feelings of Poles towards Ukrainians during the war is an interesting issue that shows subjective opinions about the existential situation perceived in the space of mutual personal interactions. The results showing opinions on help were collected from a group of 1012 people throughout Poland with the help of an authorial questionnaire. The obtained data shows a positive attitude of Poles to Ukrainians. The most willing to help are people over 49 years old, more often with a good and very good financial situation. Respondents believe that general assistance from other countries and the European Parliament is not sufficient. Military support is accepted the most by the oldest participants of research, and less by the age group up to 30 years who support medical help more. More concerned about the ongoing conflict are respondents under the age of 30 and the least wealthy, including, more often, women. The richest respondents are least afraid of the effects of the ongoing Russian–Ukrainian conflict. The conducted research confirms the openness of Polish society to refugees and brings opinions about existential solidarity with the suffering Ukrainian nation.

## 1. Introduction

The influence of armed conflict on the well-being of the population affected by the war has attracted the attention of scientists from various fields [1,2,3,4]. Some researchers indicate the economic effects of the war [5], others emphasize the tragic effects of the conflict for children [6]. So far, there is no research on subjective feelings of the population remaining outside the conflict zone, inter alia, in terms of physical, social, mental and spiritual commitment affecting the overall view of the population not being a conflict point. A sense of human safety in the hierarchy of needs occupies one of the superior places. Such values as life in dignity, stabilization and freedom away from the risk of military intervention allow members of the community to use their democratic rights. The effects of each conflict are social and cultural changes that are a multidimensional phenomenon, because they may relate to a man at every stage of development, depending on the period in which they appear, and they can take different forms. Experiences related to uncertainty and a lack of sense of security are conditioned by socio-demographic and psychological factors and affect the thoughts [7].

The population from the area covered by Russian–Ukrainian war has adapted to the situation and produced new strategies of social life and other adaptation mechanisms. Social and cultural standards have been created and will be forwarded to the next generations [8,9]. War is an event that causes strong emotions, influences views, the identity and imagination of the future, even for people not living in the area of the conflict [10]. According to Butler, war images are made available by the media, and show the fragility of life and the state of peace. Politicians and citizens of many countries have followed the events in Ukraine, trying to predict their direction and influence of the world, since April 2014, or the Russian–Ukrainian conflict in the Crimea [11,12]. In the longer term, 8 years later, on 24 February 2022, Russia’s invasion of Ukraine began [13] and showed that people involved in the event may perceive it differently than those who do not participate in it [13]. In search of a context for the contemporary image of Ukraine and Russia in the eyes of Poles, you have to consider history and think about neighboring nations not only in the historical context [14]; it is not always justified, because subjective feelings of people are subject to self-interpretation, dependent on human beings, feelings, emotions, life experiences, knowledge and items, and these can be diverse. Based on the overview of the literature, the term by researchers is most often recognized as a definition index of attitudes, feelings, political beliefs, as well as stereotypes and prejudices that are varied, and the reasons for these differences have other sources [15].

The study conducted by CBOS (2022) confirmed the great interest of Polish society, showing that the significant majority of Poles feel a fear that the conflict in Ukraine may spread and threaten Poland’s security [16]. War is an event that causes subjective feelings, usually a sense of chaos and uncertainty; it affects the emotional sphere as well as everyday life, although it does not always apply to the parties directly participating in the conflict and living in the areas covered by fights. Communities jointly discuss the current information in the context of their ideas about the future that they have made decisions on [17]. Opposition to the war does not require detailed information about who died, it becomes important by who and where they were attacked, as well as fear of their relatives. In the media, photos of anonymous victims, destroyed buildings, hospitals, schools and the civilian population were widely shown. It should be added that people confirm their ideas and feelings based on information from various sources [18,19]. The undertaken research problem is extremely important from the existential point of view and for interpersonal solidarity. Safety, health, and a dignified life for Ukrainians became the principium for generating aid attitudes among Poles. A holistic view of human existence and broadly understood empathy have characterized Polish society since the beginning of the war. It should be added that the review of the literature on the Russian–Ukrainian war presents such problems as the systematization of Polish legal acts, enabling additional assistance to Ukrainian refugees [20], threats and new challenges, the organization of medical assistance and the implications of public health for the refugee population (study based on Health Service documents statistical data) [21,22], and religious and spiritual counseling [23]. The mental health burden of the Russian–Ukrainian war, in particular the level of anxiety and depression among young adults in Central Europe has also been examined [24]. The humanitarian, economic, and financial consequences have been measured in view of the ongoing war [20], as well as the prospects and challenges related to migration to Poland [25]. The focus has also been on the problems describing the immediate threat to ecosystems, and more specifically soil degradation and food production [26,27] and the disruption of international trade, [28]. The research undertaken shows a new (real) space for helping Poles to act towards Ukrainian refugees. A significant research sample (1012 respondents from all over Poland) clearly presents the opinion captured at the beginning of the conflict. It should be emphasized that the undertaken research problem is undoubtedly important for the entire human community, especially since the direct existential aid to Ukrainians was provided by Polish society to the greatest extent. The study problem focuses on examining the subjective feelings of Poles towards Ukrainians in the era of the Russian–Ukrainian War. The set of subjective opinions seems to be a very valuable source of information that illustrates the authentic attitude of respondents. The publication presents existential behaviors and a very empathic attitude and human solidarity with the suffering Ukrainian nation.

## 2. Materials and Methods

The material was collected using a questionnaire which contained questions based on the definition of health, including the following factors: physical, mental (emotional and mental), social, and spiritual. The questionnaire was made available in electronic form on social media throughout Poland. The limitations of the study are: no access to the Internet, capturing the subjective feelings of respondents, measurement at a given moment. The main purpose of the research was to collect opinions on the need to provide humanitarian aid to refugees from the areas affected by the Russian–Ukrainian war.

A total of 1012 people took part in the survey, Table 1. The average age of respondents was 37.76 years; for women 38.89 years and men 35.27 years. Women among respondents accounted for 68.8% and men 31.2%. People under the age of 30 were 30.8% of respondents, 30–49 were 51.2% and over 49 were 18% of respondents. The education of the respondents was as follows: higher, 73.3%, medium, 24.1%, professional, 1.3%, and basic, 1.3%. Single marital status accounted for 36% of respondents, and people in a married or partner relationship made up 64% of respondents. Those in very good financial situation constituted 16.6% of respondents, good 47.6%, average 32.7%, and bad 3.1%. Village inhabitants constituted of 17.5% of respondents, the remaining 82.5% were the inhabitants of cities. Respondents living in border districts (Podlasie, Lubelskie, and Podkarpackie) accounted for 11% of all respondents. Persons without children taking part in a survey accounted for 43.6%, with those with 1 or 2 children accounting for 46.1%, and more than 2 10.3% of respondents. The questionnaire was prepared on the basis of a holistic definition of health emphasizing the state of physical, mental, and social (including the spiritual factor) human well-being [29]. The inspiration was also the theory of salutogenesis, which corresponds to the factors of the definition of health [30].

The questionnaire has been divided into 4 modules. Each of the modules concerned the following questions: Module 1. Does the interviewee support the Ukrainian nation? Module 2. Should Poles support Ukraine? Module 3. Is the help and support for Ukraine sufficient? Module 4. Do you have fears of the war in Ukraine? Module 1 included 9 questions on support of the Ukrainian nation by the respondent. People were asked about direct material help for refugees (e.g., providing a flat, donations for help for refugees, volunteering, assistance in transport, participation in the organization of fundraising, etc.), as well as non-material help, e.g., talking to refugees and showing their own support. Module 2 consisted of 5 questions concerning the respondents’ opinions about the need to support the Ukrainian nation, such as questions on whether the respondent thought that Poles should support Ukraine through first aid material, items for war purposes, articles for babies, medical supplies, or donating to charity organizations, etc. Module 3 consisted of 7 questions. The respondents answered whether they believed that the help and support offered by people and governments from Poland and abroad for Ukraine was sufficient. Module 4 included 6 questions about the respondents’ fears of the effects of the ongoing war. The questions concerned concerns about safety, economic situation, access to health care, etc. Cronbach’s Alpha’s match values for the four modules are: Module 1, 0.71, Module 2, 0.74, Module 3, 0.78, and Module 4, 0.7, so general credibility of individual questionnaire modules has been achieved. The result of the response in the modules is the value between 0 to 1. Values from 0 to 1 are interpreted within the limits from the statement “No” to “Yes”, respectively. On the other hand, the value in the middle of the range has an interpretation of “I have no opinion” for modules 2 and 3, and “medium” for modules 1 and 4. To measure the credibility of the scale of the survey a measure of Cronbach’s Alpha was used. Quantitative data is given in the form of a mean ± standard deviation, median, and percentage values. The one-sample Kolmogorov–Smirnov test showed that none of the analyzed schedules was close to normal distribution (*p* < 0.05), which is why Mann–Whitney and Kruskal–Wallis tests were used to analyze the differences between the decompositions. A Pearson correlation coefficient was used to investigate the correlation between modules. The significance level of 0.05 was adopted for all tests.

## 3. Results

**Figure 1:**
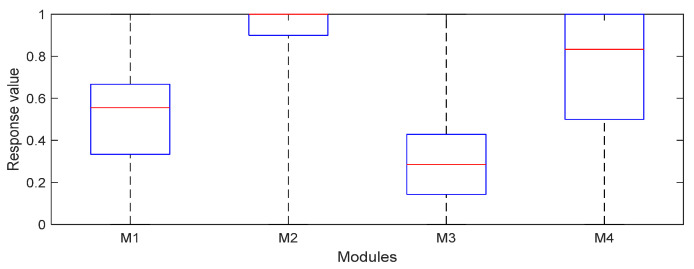
Statistics of respondents’ answers for individual modules: median, quantiles, minimum, and maximum value.

The answers from module 1 (whether the interviewee supports the Ukrainian nation) show Poles’ support is more than average. Indicators for this question, such as arithmetic mean (mean ± SD = 0.52 ± 0.23) and the median (median QD = 0.56 ± 0.17), indicate the value above 0.5. On the other hand, the values of the lower and upper quantiles indicate that 50% of respondents gave a response from 0.33 to 0.667. In the case of module 2 (whether Poles should support Ukraine) respondents’ responses were positive. Statistical indicators such as mean (mean ± SD = 0.91 ± 0.18) and the median (median ± QD = 1 ± 0.05) were close to 1. In addition, the quantile values indicate that 50% of the respondents gave a response in the range 0.9 to 1. Data obtained in 3 (if assistance and support for Ukraine are sufficient) indicates that 50% of the response of participants of the studies were distributed between 0.14 and 0.43. The average value (mean ± SD = 0.33 ± 0.23) and the median (median QD = 0.29 ± 0.14) indicate that the respondents believe that support and assistance for Ukraine are low. Answers for module 4 (do you have fears of the war in Ukraine?) confirm that the fears of respondents are high. Mean and median indicate a high level of concerns surveyed. In addition, the quantile value (Q0.25–Q0.75 = 0.5–1) indicates high fears before the war in Ukraine. In Table 2 and Figure 1, detailed statistics of responses to modules M1–M4 are presented.

The linear correlation between individual modules was also analyzed. The average positive correlation obtained for M1 and M2 modules was R = 0.4782, this correlation is statistically significant *p* < 0.001. With the increase in the support of the Ukrainian nation, the sense of respondents that this help is needed grows. The correlation coefficient showed a statistically significant negative correlation for the M1 and M3 modules (R = 0.33328, *p* < 0.001) and M2 and M3 modules (r = −0.3432, *p* < 0.001). With the increase in the support of the Ukrainian nation by respondents and the increase in the feeling that help for Ukraine is needed, the feeling of respondents that the aid and support of Ukraine are sufficient decreases. A low but statistically important correlation was received when examining the response and the age of respondents.

A Pearson correlation coefficient indicates a low positive correlation of answers to M1, M2, and M4 and age. Correlation coefficient for the age and M1 is r = 0.1328 (*p* < 0.001), for the age and M2 r = 0.0815 (*p* = 0.0095), and for the age and M4 r = 0.2091 (*p* < 0.001). For the age and M3 module, a statistically negative low linear correlation was obtained, r = −0.1623, *p* < 0.001. To examine the relationship between responses and geographic location, a Mann–Whitney test was carried out. The group was divided into two parts: the first part being the eastern border voivodships (Podlasie, Lubelskie, and Podkarpackie), and the second group was the remaining voivodships. The test showed a statistically significant difference in responses to the question about the concerns of respondents before the ongoing war in Ukraine (*p* = 0.0235). A higher average value was obtained for residents of border voivodships (mean = 0.77 ± 0.24) than for residents of others (mean = 0.71 ± 0.27). Fear of the war was stronger in border voivodships. For other questions, a Mann–Whitney test did not show differences in the responses due to the geographic location; the results are shown in Table 3.

Analyzing data split into the gender of respondents, a Mann–Whitney test assuming the significance level 0.5 showed that there was a statistically significant difference (*p* <0.001) between the answers of women and men for questions about the concern for responding before the ongoing war in Ukraine (module 4). The fears of women were significantly statistically higher than for men. Average response value and median for women were a mean ± SD = 0.76 ± 0.25, median ± QD = 0.83 ± 0.17, while in the case of men, these statistics were lower and amount to mean ± SD = 0.62 ± 0.28 and median QD = 0.67 ± 0.25. For the other three modules, a Mann–Whitney test did not show statistically significant differences between the answers of women and men. Table 3 shows statistics and results of statistical tests regarding the questionnaire responses, broken down into the respondent gender. By studying the differences between responses due to the respondent’s residence, a Mann–Whitney test did not show statistically significant differences; the results are presented in Table 3.

Considering the results of the survey in terms of respondent’s education, statistically significant differences using a Mann–Whitney test were obtained in responses to modules 1, 2, and 4. For the response of module 1 (*p* = 0.0085) on the support of the Ukrainian nation by the respondents, the group of people with higher education obtained a mean and median (mean ± SD = 0.53 ± 0.23, median ± QD = 0.56 ± 0.11) higher than for people with an education other than higher (mean ± SD = 0.49 ± 0.24 and median ± QD = 0.44 ± 0.17). A similar situation occurred for modules 2 and 4. In module 2, for a question asking if Poles should support Ukraine, both groups believed that this aid is needed. A Mann–Whitney test showed a statistically significant difference between the answers of the group of people with higher education and others (*p* = 0.0068). For a group of people with higher education, this indicator was higher (mean ± SD = 0.91 ± 0.18) than for the remaining group of respondents (mean ± SD = 0.89 ± 0.16).

Analyzing the question about the fear of war in Ukraine (module 4) a Mann–Whitney test also indicated statistically significant differences between the respondents of the group with higher education and others (*p* < 0.001). The average values and the median of respondent response ratio were higher for a group with a higher education (mean ± SD = 0.73 ± 0.26, median QD = 0.83 ± 0.25) than for other groups of people. This means that fear of the ongoing war was higher in the case of higher education than other people taking part in the survey. Detailed test results and statistics are presented in Table 3. Analyzing the response due to the age of respondents for each of the analyzed Kruskal–Wallis modules, one or more answers for each age group differed significantly (*p* < 0.05).

Responses were divided into three age groups of respondents: <30 years old, 30–49 years old and >49 years old. In the question of module 1 of the survey (supporting the Ukrainian nation) the average response rate increased with the age of respondents. The smallest support indicated the youngest respondents (below 30 years old); in this group mean and median were the lowest: mean ± SD = 0.48 ± 0.23 and median ± QD = 0.44 ± 0.17. A similar situation occurred for the responses of modules 2 and 4. In the question asking if helping Ukraine is sufficient (module 3), the indicator average value of respondents’ answers decreased, with an increase in the group below 30 years old (mean ± SD = 0.37 ± 0.23), while in the group above 49 years old, it has a mean ± SD = 0.28 ± 0.20; the median values in these cases are equal. Detailed test results are presented in Table 4.

Analyzing the response in terms of the financial situation of respondents for questions about the support of the Ukrainian nation (Module 1) a Kruskal–Wallis test showed that in one or more groups there were significant differences (*p* < 0.0001). Mean and median indicators were above 0.5 for good or very good financial situations, while they were below 0.5 for medium or bad financial situations. A similar situation occurs when asking if Poles should help Ukraine (module 2). A Kruskal–Wallis test, in this case, showed a statistically significant difference between the answers for one or more groups (*p* = 0.0082). A higher average indicator was obtained for respondents with very good and good financial situations (mean = 0.92) than for respondents with medium and bad financial situations (mean = 0.89). Additionally, for the question about fear of war in Ukraine, statistically significant differences in the answers between the analyzed groups (*p* = 0.0257) were obtained. Fear increased with a decrease in the financial situation of respondents. The lowest fear of war in Ukraine was among people with a very good financial situation, and the highest was among those with a bad financial situation. For answers to module 3, there was no statistically significant difference due to the financial situation of respondents (*p* = 0.1488), Table 4. An additional two questions were asked about the attitude to the Ukrainian nation.

The responses of the respondents were given on a scale 1–5, Table 5. Questions and answers had a character: Q1: What was your attitude towards Ukrainians before the outbreak of the war? Answer: 1–Very negative, 2–Negative, 3–Neutral, 4-positive, 5–Very positive. Q2: How has your attitude towards Ukrainians changed in the last week? Answer: 1–It has deteriorated significantly, 2–It has deteriorated on average, 3–It has not changed, 4–It has improved on average, 5–It has improved significantly. The average response result from the questionnaire for Q1 was mean = 3.36, therefore the attitude towards Ukrainians before the outbreak of the war was evaluated as neutral towards the positive direction. Based on the quantiles of half the respondents, a value of 3–4 therefore confirmed that the attitude of respondents to the Ukrainian nation is neutral or positive. For Q2, we can say that the attitude to the Ukrainian nation in recent weeks has been released or remained unchanged. The average value for Q2 questions was mean = 3.58. Low and upper quantiles have a value of 3 and 4, respectively, so 50% of respondents reported that their attitude to Ukrainians had improved on average or has not changed recently.

**Figure 2:**
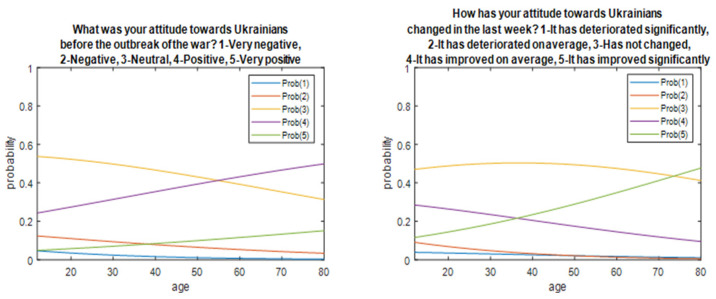
Probability distributions obtained from logistic regression concerning respondents’ answers depending on age.

In Figure 2 were presented the distributions of probability of responses 1–5.

To analyze the dependence of respondents to questions Q1 and Q2 from gender, the place of residence and geographic location, education, age, and financial situation were used in a Mann–Whitney test or a Kruskal–Wallis test. A Mann–Whitney test showed a statistically significant difference between the answers of women and men to Q2 (*p* = 0.0075). The value of the statistics for men (mean ± SD = 3.68 ± 0.18) was higher than for women (mean ± SD = 3.53 ± 0.96), therefore the attitude to the Ukrainian nation in the case of men had a greater change than among women in recent weeks. A Kruskal–Wallis test showed that there were statistically significant differences between answers in various age groups and groups of various financial situations in each of these cases for questions Q1 and Q2 *p* < 0.01.

Therefore, it can be concluded that the attitude to the Ukrainian nation among the surveyed is better among people over 49 (mean ± SD = 3.53 ± 0.77) than among younger people. Changing the income after the outbreak of the war in a positive direction is higher among people over 49 years (mean ± SD = 3.87 ± 0.99) than among younger respondents. Considering the financial situation of respondents, the attitude of the surveyed to the Ukrainian nation is positive among people with a very good financial situation (mean ± SD = 3.47 ± 0.8). Additionally, changing the attitude to the Ukrainian nation after the outbreak of the war in Ukraine has strongly improved among people with a very good financial situation (mean ± SD = 3.76 ± 0.93) than other respondents. In other cases, the tests did not show statistically significant differences in respondents’ replies, Table 6 and Table 7.

## 4. Discussion

Research results indicate that the attitude of Poles towards Ukrainians is generally positive. Respondents believe that the Ukrainian nation should be supported in their misfortune. To illustrate the obtained data, it should be noted that the answers to the questions are ordered in 4 modules. In the first part (module) of the survey, respondents were asked about supporting the Ukrainian nation and how they join in the help of refugees. The respondents answered whether they help directly through the provision of a flat, or assist in transporting population, foodstuffs, industrial goods, or medicines. They were asked if they participate in volunteering, direct interactions, or organizing money collections. They were also asked to express opinions on supporting assistance and subjective feelings. The data obtained indicate that the Polish society significantly supports the Ukrainian nation and expresses its empathy for those needing help. Moreover, along with the increase in declared help by respondents, the feeling is also a sense that this aid is essential.

At the same time, the awareness of the generation of aid attitude increases with the age of respondents. The least involved in support are respondents up to 30 years. It should be added that refugees can count on a lot of help from Poles with a good and very good material situation, and people with higher education are ready to support the most. The above behavior of respondents corresponds to other research results, which indicate that people with a higher level of humanitarianism are more willing to accept refugees escaping from natural disasters and wars than those who flee before political repression [31]. As opposing behaviors, one can recall the attitudes of respondents expressed in xenophobic demonstrations, assaults, arsons, and different attacks on the residence of refugees in Germany in 2014–2015 [32]. Collected data in the second module of the survey allowed us to obtain the answer to if Poles should support Ukraine by purchasing military equipment (bulletproof vests, night vision, binoculars, thermovisers), as well as sleeping bags, inflatable mattresses, and sleeping pads. It was also asked whether Poles should support by providing medical articles (bandages, painkillers, oxidized water, patches) and articles for children and babies (food and care). They were also asked to express an opinion on cash payments to organizations that help Ukraine.

The obtained answers indicate that participants of research with higher education are ready to support the Ukrainian population. At the same time, the research shows that the respondents with a good and very good financial situation will provide this help more willingly. In addition, it should be added that respondents who decide to help and are convinced that this aid is obligatory believe that general assistance from other countries is not sufficient. Respondents up to the age of 30 least support military aid, which may indicate that young people are against the armed resolution of the conflict. The youngest respondents are determined to help by providing medical and care agents to the aggrieved parties. Providing help to women in advanced pregnancy with chronic diseases and children with mental problems and with educational deficits is a form of humanitarianism that Polish society is involved in.

Empathic references of young respondents from the perspective of medical assistance can confirm the results of other studies that show a presentation of refugees in the media, where images of small groups of people with visible faces generate aid and pro-humanitarian actions. On the other hand, images of large groups are evaluated as more reluctant, especially in combination with metadata, for example, headers (titles) from newspapers [33]. In the third module, respondents had to answer whether general material assistance and military support from Poland and the whole world was sufficient and were asked to express opinions on aid decisions made by the European Parliament and the European Union, as well as restrictions taken against Russia. According to the answers granted by the participants of research, it can be concluded that support for Ukraine is low and insufficient. At the same time, the oldest participants of research believe that aid is needed, and the youngest that it is not enough.

The position of the refugees described in the literature is a multi-faceted problem and puts them as people who need comprehensive support [34]. Researchers show that a global attitude towards refugees or other victims of global disasters is noticeable, especially among those who are far from the disaster zone [35]. In the last part (module 4) respondents disclosed concerns about the ongoing war. They were asked if they feel internal anxiety related to the Russian–Ukrainian conflict and if it is a direct threat to Poland in the form of an active assault. In addition, the respondents expressed their opinion on the deterioration of their state of health and access to public health protection. The answers indicate the anxiety of Polish society against the threats of the ongoing war. The most concerned about this situation are respondents under the age of 30 and the least wealthy. At the same time, the most affluent participants of the research are least afraid of the effects of the ongoing Russian–Ukrainian conflict.

The results from the conducted research also indicate that respondents living in border areas (Podlasie, Lubuskie, and Podkarpackie) show greater anxiety. Such a state indicates a sense of existential discomfort of the inhabitants of border areas and with areas where the armed conflict is pending. On the basis of the conducted research, there can be no statistically drawn differences in women and men towards the Russian–Ukrainian war. The greatest discrepancies can be noticed in answers to questions from the fourth module, including questions about fears against the ongoing war, where women’s fears are greater than men’s. In the other three modules, women’s and men’s responses are convergent. Based on the data obtained, one can observe the current attitude of Poles to Ukrainians, being in a very difficult existential situation. The obtained data indicate that the attitude of Polish society towards Ukrainians is neutral or positive, and in recent weeks improved or remains unchanged. At the same time, the adjustment of 50% of respondents has been released or changed. The attitude towards Ukrainians in recent weeks has significantly improved among men. Taking into account the age of respondents, the largest change of attitudes took place in people over 49 and the most prosperous. It is worth noting that women and children constitute the largest group of refugees from Ukraine, which may affect the positive attitude of Polish society.

Considering the above attitudes, the results indicate a positive attitude towards children who generate similar behaviors in adults [36]. However, reports from research on immigrants from France show that the natives there, who sporadically contact immigrants, are less accommodating of immigrants from non-Western countries, and more accommodating to newcomers from Western countries. In turn, the natives who conduct frequent social interactions with refugees attach less importance to nationality as a criterion for admission of immigrants [37]. Swiss scientists, on the basis of the conducted research, came to the conclusion that the participation of immigrants who are culturally different is a significant and considerable determinant of antimigration votes, while the presence of immigrants who are culturally similar does not affects the attitudes of respondents at all [38].

However, in the literature you can find opinions suggesting that supportive attitudes are less positive, for example, to refugees from Muslim countries [39]. Summing up, it should be noted that Ukrainians staying in Poland are perceived positively. However, considering the space of various negative experiences during the war, emotional problems should be expected, generating a sense of fear among young people (minors). The conducted research in this matter indicates the need for social support, which best favors the processes of adaptation to new living conditions [40]. It should be emphasized that research confirms negative psychosocial states in terms of fear and depressive states of people who have experienced physical violence, especially rape. The authors emphasize that the lack of social support for these people is a predictor of high anxiety and post-traumatic stress [41].

Researchers believe that aid interactions towards people who have experienced violence during the war should be trusted, which is necessary to build a form of verification in the actions taken [42]. In addition, specialists suggest that social workers apply methods of engaging in direct interactions to maintain a supportive and safe environment for participants of hostilities, e.g., in the field of education. Persons affected by the trauma should be able to build their strengths and increase psychological resources to ensure personal welfare [43]. According to data from 2017, nearly 66 million people resettled as a result of armed conflicts, including a few million war refugees from Ukraine. Research carried out in terms of mental support indicates a significant lack of data on the wider scope of psychiatric disability among people living in prolonged resettlement situations. Effective tools are needed to support the implementation of a balanced global mental health policy in the countries torn by war and intervention in the area of mental support of refugees [44].

## 5. Conclusions

The results from the conducted research show a positive attitude of Poles towards Ukrainians in the era of the Russian–Ukrainian War. The most willing to help are respondents over 49 years of age. Respondents with a good and very good financial situation are more willing to help refugees. Poles who want to help, and have an internal conviction that this help flows out of human solidarity, believe that general assistance from other countries and the European Parliament is not sufficient. The oldest participants of the research believe that aid is necessary, and the youngest participants have a different opinion. Military support is accepted the most by the oldest participants of research, and less by the age group up to 30, who are more open to medical assistance. The respondents under the age of 30 and the least wealthy are concerned about the ongoing armed conflict. The most affluent materially feel the least fear of the ongoing Russian–Ukrainian conflict. Humanitarian opinions of Poles reflect authentic assistance attitudes towards Ukrainians in need of existential versatile help. The collected opinions present the subjective opinions of the research participants, which, from a strictly scientific point of view, are not entirely an objective picture of reality, but show the emotional and existential opinions of the respondents measured from physical, psychological, and social perspectives in a specific time and existential circumstance.

## Figures and Tables

**Table 1:** The number of respondents and the average age according to the division into groups.

			Age	Age
Group of respondents	N	%	Mean ± SD	Median ± QD
All respondents	1012	100	37.76 ± 12.75	37 ± 9.5
Women	696	68.8	38.89 ± 12.45	39 ± 9
Men	316	31.2	35.27 ± 13.05	33 ± 10
Age < 30 years old	312	30.8	23.28 ± 3.43	23 ± 3
Age 30–49 years old	518	51.2	39.58 ± 5.54	40 ± 4.5
Age < 49 years old	182	18.0	57.37 ± 6.48	56 ± 5
City	835	82.5	37.71 ± 12.68	37 ± 9.5
Village	177	17.5	37.98 ± 13.11	38 ± 10.5

**Table 2:** Statistics of respondents’ answers for individual modules.

All Respondents					
Module	N	Mean ± SD	Median ± QD	Q_0.25_–Q_0.75_	Min–max
M1	1012	0.52 ± 0.23	0.56 ± 0.17	0.33–0.67	0–1
M2	1012	0.91 ± 0.18	1 ± 0.05	0.9–1	0–1
M3	1012	0.33 ± 0.23	0.29 ± 0.14	0.14–0.43	0–1
M4	1012	0.71 ± 0.27	0.83 ± 0.25	0.5–1	0–1

**Table 3:** Comparison of the answer of the given question (module M1–M4) with geographic location, gender, place of residence, and education.

					Mann–Whitney Test	
Module	Variable	Mean ± SD	Median ± QD	N	z-Value	*p*
	Geographic location					
M1	Eastern border voivodships	0.5 ± 0.23	0.44 ± 0.17	113	−1.2311	0.2183
	Other	0.52 ± 0.23	0.56 ± 0.17	899		
M2	Eastern border voivodships	0.9 ± 0.20	1 ± 0.1	113	0.2903	0.7716
	Other	0.91 ± 0.18	1 ± 0.05	899		
M3	Eastern border voivodships	0.35 ± 0.20	0.29 ± 0.11	113	1.7616	0.0781
	Other	0.32 ± 0.23	0.29 ± 0.14	899		
M4	Eastern border voivodships	0.77 ± 0.24	0.83 ± 0.17	113	2.2647	0.0235
	Other	0.71 ± 0.27	0.83 ± 0.25	899		
	Gender					
M1	Women	0.52 ± 0.23	0.56 ± 0.11	696	1.2619	0.2070
	Men	0.50 ± 0.24	0.56 ± 0.17	316		
M2	Women	0.91 ± 0.17	1 ± 0.05	696	−1.0600	0.2892
	Men	0.91 ± 0.18	1 ± 0.05	316		
M3	Women	0.32 ± 0.23	0.29 ± 0.14	696	−0.4191	0.6751
	Men	0.33 ± 0.24	0.29 ± 0.14	316		
M4	Women	0.76 ± 0.25	0.83 ± 0.17	696	7.2007	<0.001
	Men	0.62 ± 0.28	0.67 ± 0.25	316		
	Place of residence					
M1	City	0.51 ± 0.23	0.56 ± 0.17	835	−1.5107	0.1309
	Village	0.55 ± 0.23	0.56 ± 0.11	177		
M2	City	0.9 ± 0.19	1 ± 0.05	835	−1.4820	0.1383
	Village	0.93 ± 0.13	1 ± 0.05	177		
M3	City	0.33 ± 0.24	0.29 ± 0.14	835	1.4486	0.1474
	Village	0.29 ± 0.20	0.29 ± 0.14	177		
M4	City	0.71 ± 0.27	0.83 ± 0.17	835	−0.5551	0.5789
	Village	0.72 ± 0.27	0.83 ± 0.25	177		
	Education					
M1	Higher	0.53 ± 0.23	0.56 ± 0.11	742	2.6302	0.0085
	Other	0.49 ± 0.24	0.44 ± 0.17	272		
M2	Higher	0.91 ± 0.18	1 ± 0.05	742	2.7047	0.0068
	Other	0.89 ± 0.16	1 ± 0.1	270		
M3	Higher	0.32 ± 0.23	0.29 ± 0.14	742	−1.5092	0.1313
	Other	0.34 ± 0.23	0.29 ± 0.18	270		
M4	Higher	0.73 ± 0.26	0.83 ± 0.25	742	3.5883	<0.001
	Other	0.66 ± 0.30	0.67 ± 0.25	270		

**Table 4:** Comparison of the answer of the given question (module M1–M4) with age and financial situation.

					Kruskal–Wallis Test		
Module	Variable	Mean ± SD	Median ± QD	N	Chi-Sq	df	*p*
	Age						
M1	<30 years old	0.48 ± 0.23	0.44 ± 0.17	312	13.9150	2	<0.0001
	30–49 years old	0.52 ± 0.23	0.56 ± 0.11	518			
	>49 years old	0.55 ± 0.21	0.56 ± 0.11	182			
M2	<30 years old	0.9 ± 0.18	1 ± 0.1	312	11.4715	2	0.0032
	30–49 years old	0.91 ± 0.19	1 ± 0.5	518			
	>49 years old	0.93 ± 0.15	1 ± 0.5	182			
M3	<30 years old	0.37 ± 0.23	0.29 ± 0.14	312	23.3599	2	<0.0001
	30–49 years old	0.31 ± 0.24	0.29 ± 0.14	518			
	>49 years old	0.28 ± 0.20	0.29 ± 0.14	182			
M4	<30 years old	0.63 ± 0.29	0.67 ± 0.25	312	41.4651	2	<0.0001
	30–49 years old	0.74 ± 0.25	0.83 ± 0.17	518			
	>49 years old	0.78 ± 0.25	0.83 ± 0.17	182			
	Financial situation						
M1	Very good	0.57 ± 0.22	0.56 ± 0.14	168	34.4788	3	<0.0001
	Good	0.54 ± 0.22	0.56 ± 0.11	482			
	Average	0.47 ± 0.24	0.44 ± 0.17	331			
	Bad	0.42 ± 0.27	0.44 ± 0.22	31			
M2	Very good	0.92 ± 0.18	1 ± 0.05	168	11.7815	3	0.0082
	Good	0.92 ± 0.16	1 ± 0.05	482			
	Average	0.89 ± 0.20	1 ± 0.1	331			
	Bad	0.89 ± 0.17	1 ± 0.1	31			
M3	Very good	0.34 ± 0.25	0.29 ± 0.16	168	5.3353	3	0.1488
	Good	0.31 ± 0.22	0.29 ± 0.14	482			
	Average	0.34 ± 0.23	0.29 ± 0.16	331			
	Bad	0.35 ± 0.26	0.29 ± 0.20	31			
M4	Very good	0.68 ± 0.29	0.67 ± 0.25	168	9.2901	3	0.0257
	Good	0.70 ± 0.27	0.75 ± 0.25	482			
	Average	0.74 ± 0.26	0.83 ± 0.17	331			
	Bad	0.76 ± 0.27	0.83 ± 0.25	31			

**Table 5:** Statistics of respondents’ answers for questions Q1 and Q2.

All Respondents					
Question	N	Mean ± SD	Median ± QD	Q_0.25_–Q_0.75_	Min–max
Q1	1012	3.36 ± 0.83	3 ± 0.5	3–4	1–5
Q2	1012	3.58 ± 0.97	3 ± 0.5	3–4	1–5

**Table 6:** Comparison of the answer of the given question (Q1, Q2) with geographic location, gender, place of residence, and education.

					Mann–Whitney Test	
Question	Variable	Mean ± SD	Median ± QD	N	z-Value	*p*
	Geographic location					
Q1	Eastern border voivodships	3.37 ± 0.78	3 ± 0.5	113	0.08	0.9362
	Other	3.39 ± 0.83	3 ± 0.5	899		
Q2	Eastern border voivodships	3.47 ± 1.01	3 ± 0.5	113	−0.9141	0.3607
	Other	3.59 ± 0.96	3 ± 0.5	899		
	Gender					
Q1	Women	3.41 ± 0.79	3 ± 0.5	696	1.4637	0.1433
	Men	3.33 ± 0.9	3 ± 0.5	316		
Q2	Women	3.53 ± 0.96	3 ± 0.5	696	−2.6723	0.0075
	Men	3.68 ± 0.18	4 ± 1	316		
	Place of residence					
Q1	City	3.37 ± 0.84	3 ± 0.5	835	−1.5433	0.1228
	Village	3.48 ± 0.76	3 ± 0.5	177		
Q2	City	3.56 ± 0.97	3 ± 0.5	835	−1.1318	0.2577
	Village	3.66 ± 0.96	3 ± 1	177		
	Education					
Q1	Higher	3.41 ± 0.84	3 ± 0.5	742	1.6523	0.0985
	Other	3.32 ± 0.80	3 ± 0.5	272		
Q2	Higher	3.55 ± 0.97	3 ± 0.5	742	−1.3549	0.1754
	Other	3.64 ± 0.97	3 ± 0.5	270		

**Table 7:** Comparison of the answer of the given question (Q1, Q2) with age and financial situation.

					Kruskal–Wallis Test		
Question	Variable	Mean ± SD	Median ± QD	N	Chi-Sq	df	*p*
	Age						
Q1	<30 years old	3.22 ± 0.81	3 ± 0.5	312	20.7022	2	<0.0001
	30–49 years old	3.44 ± 0.85	3 ± 0.5	518			
	>49 years old	3.53 ± 0.77	3.5 ± 0.5	182			
Q2	<30 years old	3.52 ± 0.98	3.5 ± 0.5	312	19.4170	2	<0.0001
	30–49 years old	3.51 ± 0.94	3.5 ± 0.5	518			
	>49 years old	3.87 ± 0.99	4 ± 1	182			
	Financial situation						
Q1	Very good	3.47 ± 0.8	3 ± 0.5	168	11.5827	3	0.0090
	Good	3.45 ± 0.82	3 ± 0.5	482			
	Average	3.27 ± 0.84	3 ± 0.5	331			
	Bad	3.16 ± 0.9	3 ± 0.5	31			
Q2	Very good	3.76 ± 0.93	4 ± 1	168	13.4497	3	0.0038
	Good	3.62 ± 0.97	3 ± 0.5	482			
	Average	3.45 ± 0.97	3 ± 0.5	331			
	Bad	3.32 ± 0.98	3 ± 0.5	31			

## Data Availability

Data available on request from the authors.
